# 

**Published:** 1923-10

**Authors:** 


### DIRECTORY

FOR

### Members of the Dental Profession whose practices are limited to a specialty.

The object of this directory is to furnish ready reference to specialists who take referred patients. If you wish your name inserted in this list please correspond with the Dental Register, Box 838, Cincinnati, Ohio.

Your name, specialty, office hours and phone number will be published.

This entire page is reserved for Professional Directory.

### Cincinnati

Dental X-Ray Diagnosis
CHARLES K. FIELD
2709 Woodburn Ave., Walnut Hills
Hours, 10 to 4
Woodburn 2486

Periodontoclasia (Pyorrhea and Oral Prophylaxis)
EDGAR H. EBERLE
605-06 Union Central Bldg., 4th and Vine Sts.
Hours, 9 to 5—Consultation hours by appointment
Main 882

Periodontoclasia (Pyorrhea and Oral Prophylaxis)
MARIE L. BECKERT
25 Groton Bldg., 7th and Race Sts.
Hours, 9 to 5—Consultation hours by appointment
Canal 842

Exodontia (Extraction and Oral Surgery)
ROBERT M. SCHELL
20 W. 9th St., between Vine and Race Sts.
Hours, 1 to 5—Consultation by appointment
Canal 118

